# Therapeutic revolution for inoperable stage III non-small cell lung cancer in the immune era

**DOI:** 10.20892/j.issn.2095-3941.2022.0254

**Published:** 2022-06-09

**Authors:** Jiakang Li, Jingyan Xu, Mingyi Yang, Qing Zhou

**Affiliations:** 1 School of Medicine, South China University of Technology, Guangzhou 510006, China; 2 Guangdong Lung Cancer Institute, Guangdong Provincial People’s Hospital, Guangdong Academy of Medical Sciences, Guangzhou 510080, China; 3 The Second School of Clinical Medicine, Southern Medical University, Guangzhou 510000, China

## The PACIFIC study ushered in a “tsunami-like” therapeutic revolution for stage III inoperable non-small cell lung cancer (NSCLC)

In the past, chemoradiotherapy (CRT) has been the standard of care for inoperable stage III NSCLC. Concurrent chemoradiotherapy (cCRT), if tolerable in patients, is the optimal treatment regimen. A meta-analysis has shown that cCRT results in a 5-year survival rate 4.5% longer than that with sequential chemoradiotherapy (sCRT)^1^. However, within 2 years after cCRT, approximately 30% of patients experience local recurrence, and approximately 40% develop distant metastasis^2^. Clinicians have explored induction chemotherapy^3^, consolidation chemotherapy^4^, and combination use with targeted drugs^2^, and found that none improve the prognosis.

With the development of anti-PD-1/PD-L1 drugs, the PACIFIC study has revolutionized the therapeutic landscape for inoperable stage III NSCLC, in a manner that has been likened to a “tsunami”. The PACIFIC study^5^ was a randomized, double-blind phase 3 clinical study that enrolled patients with stage III NSCLC who had no progression after cCRT. These patients received consolidation therapy with durvalumab or placebo for 1 year. The primary endpoints of the study were progression-free survival (PFS) and overall survival (OS). The most recent 5-year follow-up data have shown^6^ that treatment with durvalumab increased the median OS by 18.4 months, and 42.9% of patients had an OS exceeding 5 years; the median PFS (mPFS) increased by 11.3 months [16.9 months *vs.* 5.6 months, hazard ratio (HR) = 0.55]. Approximately one-third of patients remained progression-free at 5 years. Consolidation therapy with durvalumab led to stable and sustained PFS and OS; moreover, the 5-year OS rate improved by 9.5% after cCRT, without a significant difference in adverse reactions with respect to the placebo group. This finding provided hope for a “clinical cure” of inoperable stage III NSCLC.

In the real-world study PACIFIC-R^7^, 1,399 patients received durvalumab, and the mPFS was 21.7 months (14.3% of patients received sCRT). The study showed promising efficacy and manageable safety in the real world. The PACIFIC study revealed the synergistic effect of immunotherapy and chemoradiotherapy. Durvalumab is the only immunotherapeutic drug approved for this indication worldwide, according to the Chinese Society of Clinical Oncology (CSCO) guidelines and National Comprehensive Cancer Network (NCCN) guidelines, and is the standard treatment for inoperable stage III NSCLC.

## GEMSTONE-301: The first study to introduce immunotherapy after sequential chemoradiotherapy, according to patient needs

In clinical practice, fit patients are usually selected to receive cCRT^8^. Many patients cannot tolerate cCRT and receiving cCRT may increase the incidence of adverse reactions, such as radiation pneumonitis and esophageal toxicity^9,10^. Moreover, close multidisciplinary cooperation among medical institutions is usually required for cCRT, thus hindering the implementation of cCRT in clinical practice. For patients with inoperable stage III NSCLC who cannot tolerate cCRT or are unable to receive cCRT because of medical conditions, sCRT is a well-established alternative therapy. Surveys have indicated that, although the proportion of patients receiving cCRT in the real world has increased since the publication of the PACIFIC study results, approximately 40%–70% of patients receiving CRT are treated with sCRT in clinical practice in China and several European countries^11,12^. The GEMSTONE-301 study, led by Professor Yilong Wu^13,14^, included patients with *EGFR/ALK*-negative, inoperable stage III NSCLC who had no progression after cCRT and sCRT. Patients received consolidation therapy with the anti-PD-L1 monoclonal antibody sugemalimab or placebo for 2 years. The primary endpoint was PFS. The study indicated significantly longer PFS in the sugemalimab group than the placebo group (mPFS: 9.0 months *vs.* 5.8 months, respectively, HR = 0.64, *P* = 0.0026). Treatment with sugemalimab showed a consistent PFS benefit in both the cCRT and sCRT subgroups. The OS data are not yet mature, and follow-up is ongoing. The GEMSTONE-301 study was the first to extend immunotherapeutic treatment to a population receiving sCRT; it excluded patients with *EGFR* mutation-positive, which have a high incidence in China, and extended the duration of immune consolidation therapy to 2 years. The study design considered the needs of Chinese patients, and the findings supported the rationale for immune consolidation therapy after cCRT and sCRT. On the basis of the study, sugemalimab is expected to be approved for immune consolidation therapy after CRT for inoperable stage III NSCLC by the National Medical Products Administration (NMPA) in mid-2022.

Several important differences in study design exist between GEMSTONE-301 and PACIFIC. GEMSTONE-301 included patients treated with sCRT, excluded patients with positive driver genes, and used a consolidation therapy duration of 24 months. In addition, significant differences existed in the baseline characteristics of patients. First, only 29% of patients in GEMSTONE-301 had stage IIIA disease, and most patients had stage IIIB and IIIC disease, whereas 53% had stage IIIA disease in the PACIFIC study. Second, squamous cell carcinoma accounted for 69% cases in the GEMSTONE-301 study and 46% in the PACIFIC study. Therefore, more patients with refractory disease were included in the GEMSTONE-301 study. However, the magnitude of benefit of immune consolidation therapy was similar in both studies, with HRs for PFS of 0.55 and 0.64, respectively, and a significant decrease in the risk of disease progression. These 2 large phase 3 randomized controlled clinical studies clearly established the value of immune consolidation therapy after cCRT and sCRT. The phase 3 clinical study PACIFIC-5^15^ is currently ongoing and will also explore the benefit of durvalumab in patients receiving sCRT.

## Could a major breakthrough in treatment of unresectable stage III NSCLC be achieved by applying advanced immunotherapy earlier along with targeted therapy?

In the PACIFIC and GEMSTONE-301 studies, immune consolidation therapy after CRT has been found to be successful. However, 22%–30% of patients fail to complete the full course of treatment because of disease progression or intolerable toxicity during CRT treatment, and therefore miss the opportunity to receive immune consolidation therapy^16,17^. Could immunotherapy be applied simultaneously with CRT, or even earlier in the induction phase, thereby enabling more patients to benefit from immunotherapy? KEYNOTE-799^18^, a phase 2 uncontrolled study, has enrolled treatment-naïve patients with inoperable stage III NSCLC. Patients received pembrolizumab plus chemotherapy induction therapy for one cycle, followed by pembrolizumab plus cCRT, and then pembrolizumab consolidation therapy. The regimen showed promising clinical efficacy, with a 1-year PFS rate of 67.1%–71.6%, and 1-year OS rate of 81.3%–87%. However, the incidence of grade ≥ 3 pneumonia was 6.9%–8%, and the incidence of toxicity was higher than that in the PACIFIC study, thereby suggesting that phase 3 clinical study data are required to assess the safety of pembrolizumab combined with cCRT. The ongoing phase 3 clinical study KEYLYNK-012 of pembrolizumab combined with cCRT followed by olaparib maintenance will further evaluate the risks and benefits, and is expected to clarify the appropriate target population. In addition, a phase 3 clinical trial of durvalumab combined with cCRT (PACIFIC-2) is ongoing, and phase 3 clinical studies of cCRT with immunotherapy are also being conducted for pembrolizumab (KEYLYNK-012), nivolumab (CheckMate73L), and tislelizumab (AdvanTIG-301). These studies will further assess whether new breakthroughs can be achieved in the current modality of immune consolidation therapy after chemoradiotherapy for inoperable stage III NSCLC. Immunotherapy combined with cCRT may allow more patients with stage III unresectable NSCLC to receive immunotherapy earlier than immune consolidation therapy. Phase 3 clinical data are required to further assess the toxicity and safety profile and to determine whether this treatment modality might decrease disease progression during radiotherapy and improve the local disease control rate. Doctors should consider patient tolerance, select appropriate patients, precisely delineate the radiotherapy target volume, and address adverse reactions in a timely manner to ensure the successful completion of radical treatment during triple combination therapy comprising radiotherapy, chemotherapy, and immunotherapy.

In addition to earlier immunotherapeutic intervention, combination treatment with new drugs is also expected to be a promising means to further improve the prognosis of patients with inoperable stage III NSCLC. Clinical trials of anti-PD-1/PD-L1 drugs in combination with TIGIT, CTLA-4, PARP, anti-vascular, NKG2A, and CD73 targets are ongoing. The COAST study^19^, a phase 2 multi-agent platform study, has enrolled patients with stage III unresectable NSCLC without progression after cCRT. Durvalumab plus oleclumab (anti-CD73), or durvalumab plus monalizumab (anti-NKG2A), significantly improved the objective response rate and prolonged the 10-month PFS (72.7% and 64.8%, respectively) beyond that with consolidation therapy with durvalumab alone (39.2%), without new safety concerns. The COAST study, the first study to combine a new drug based on the PACIFIC study, has shown promising clinical efficacy, thus supporting further registration of studies. Because the duration of consolidation therapy generally exceeds 1 year, anti-PD-1/PD-L1-based combination therapy should focus on the safety of long-term treatment and whether the optimal combination regimen needs to be selected according to biomarkers.

In the future, with the publication of more research results, physicians will have several treatment options for stage III inoperable NSCLC, with possibilities of clinical cure. The survival and prognosis of patients with locally advanced NSCLC has significantly improved in the era of immunotherapy. The PACIFIC and GEMSTONE-301 studies have established an immune consolidation therapy paradigm after chemoradiotherapy. Radiotherapy is the standard of care for unresectable stage III NSCLC, thus potentially inducing the expression of tumor-specific antigen peptides, or neoantigens, that clinically synergize with immune checkpoint therapy. Some tumor-specific antigen peptides or neoantigens may potentially influence the survival benefit in patients with NSCLC^20^. In the future, more patients will be able to reap the benefits of immunotherapy by combination with cCRT or new targeted therapy. For stage III NSCLC, a group of highly heterogeneous and complex diseases, physicians treating lung cancer may face problems in deciding how to choose interventional immunotherapy and the duration of immunotherapy, and whether to use combined therapy. Hence, surgeons, radiotherapists, and internists should use multidisciplinary diagnosis and treatment and whole-course management for each patient with stage III NSCLC to enable more patients to benefit from multidisciplinary treatment, and achieve higher survival rates, clinical cure, and better quality of life.
